# Sudden Infant Death Syndrome on Facebook: Qualitative Descriptive Content Analysis to Guide Prevention Efforts

**DOI:** 10.2196/18474

**Published:** 2020-07-30

**Authors:** Kelly Pretorius, Eunju Choi, Sookja Kang, Michael Mackert

**Affiliations:** 1 The University of Texas at Austin Austin, TX United States

**Keywords:** sudden infant death, SIDS, infant mortality, safe sleep, social media, social support, health communication, maternal health, qualitative research, health care providers

## Abstract

**Background:**

Sudden unexpected infant death (SUID), which includes the diagnosis of sudden infant death syndrome (SIDS), is a leading cause of infant mortality in the United States. Despite prevention efforts, many parents continue to create unsafe infant sleep environments and use potentially dangerous infant sleep and monitoring devices, ultimately leading to sleep-related infant deaths. Analyzing Facebook conversations regarding SIDS may offer a unique maternal perspective to guide future research and prevention efforts.

**Objective:**

This study aims to describe and analyze conversations among mothers engaged in discussions about SIDS on a Facebook mother’s group. We were interested in understanding maternal knowledge of SIDS, identifying information sources for SIDS, describing actual infant sleep practices, exploring opinions regarding infant sleep products and monitoring devices, and discovering evidence of provider communication regarding SIDS.

**Methods:**

We extracted and analyzed 20 posts and 912 comments from 512 mothers who participated in a specific Facebook mother’s group and engaged in conversations about SIDS. There were 2 reviewers who coded the data using qualitative descriptive content analysis. Themes were induced after discussion among researchers and after the study objectives were addressed.

**Results:**

The theme of social support emerged, specifically informational and emotional support. A variety of informational sources for SIDS and safe sleep were identified, as was a continuum of infant sleep practices (ranging from unsafe to safe sleep per the American Academy of Pediatrics standards). There was widespread discussion regarding infant sleep products and monitoring devices. Embedded within conversations were (1) confusion among commonly used medical terminology, (2) the practice of unsafe infant sleep, (3) inconsistency in provider communication about SIDS, and (4) maternal anxiety regarding SIDS.

**Conclusions:**

We uncovered new findings in this analysis, such as the commonality of infant sleep products and monitoring devices and widespread maternal anxiety regarding SIDS. Additionally, mothers who participated in the Facebook group provided and received informational and emotional support regarding SIDS via this social media format. Such results can guide future prevention efforts by informing health communication regarding SUID and safe sleep. Future provider and public health agency communication on the topic of SUID and safe sleep should be simple and clear, address infant sleep products and monitoring devices, address maternal anxiety regarding SIDS, and address the common practice of unsafe sleep.

## Introduction

### Background

Sudden unexpected infant death (SUID) and sudden infant death syndrome (SIDS) are a leading cause of infant mortality in the United States, resulting in approximately 3500 infant deaths annually [1]. SUID includes the diagnosis of SIDS and other unintentional causes of infant death: asphyxia, accidental suffocation and strangulation in bed, or ill-defined deaths. Risk factors for SUID include prone sleeping, bed sharing, soft bedding, unsafe sleep surfaces, prematurity, and smoke exposure [1]. Although the Back to Sleep campaign [2] reduced SIDS deaths by more than 50%, SIDS remains to be the leading cause of postneonatal mortality in the United States [3]. Furthermore, there has been an increase in infant deaths because of accidental suffocation [4].

Despite recommendations for SUID prevention, as of 2015, approximately 21% of mothers reported placing infants on their stomach to sleep, 61.4% reported bed sharing, and 38.5% reported using soft bedding, such as blankets or bumper pads [5]. Another study found that 54.7% of mothers reported the use of hazardous bedding for their infants [6]. Furthermore, potentially dangerous infant sleeping devices (eg, Rock ‘n Play [7], baby swings) and sleep monitoring devices (eg, Owlet, Snuza) are increasingly common, yet little is known about their use [8].

Mothers place infants to sleep in the prone position for perceived safety, infant comfort, and concern for choking [9,10]. Colson et al [11] found that mothers who were advised consistently by their doctors to practice supine sleeping were less likely to place infants prone. Mothers were also more likely to follow social norms or model behaviors that were perceived as positive [11]. Such perspectives may be influenced by cultural norms and family members [10]. Extended social networks, such as social media, are also potentially influential. Social media is available 24/7 for people to seek information or leave messages for peers [12]. Thus, mothers are increasingly using social media for parenting and health information [13,14]. As of 2015, 75% of parents used social media, often for parenting information or support [14]. In a review of parental use of social media for parenting in the United States, Facebook was the most common social media format [15]. Facebook was popular among all races or ethnicities, including African Americans [15]—those most at risk for SUID [1].

### Objectives

Thus, this study aimed to (1) understand maternal knowledge of SIDS, (2) identify information sources for SIDS, (3) describe actual infant sleep practices, (4) explore opinions regarding infant sleeping devices and sleep monitoring devices as they relate to SIDS, and (5) discover evidence of provider communication regarding SIDS among mothers engaged in discussions about SIDS on a Facebook mother’s group.

## Methods

This is a qualitative study of publicly available data extracted from a Facebook mother’s group in May 2019. At that time, there were 17,545 members in the group. Once on the Facebook page, *SIDS* was entered in the search toolbar. The results were filtered to include the following: *all posts*, *most recent*, posted by *anyone*, tagged location of *anywhere*, and *any date* posted. The phrase *safe sleep* was trialed but ultimately not selected as this resulted in erroneous conversations. The term *SIDS*, on the other hand, resulted in a total of 20 posts that were all relevant to SIDS or safe sleep. Each post and related conversations (912 comments) were manually copied and pasted into a spreadsheet. To protect the identity of the participants, only the initials of the participant were copied to the spreadsheet to enable the researchers to follow the conversations. After the posts and replies were copied, the spreadsheet was manually compared with the actual Facebook page to ensure accuracy. Personal and identifying information were removed to maintain confidentiality.

The posts and related conversations were then converted to 20 documents to be uploaded to Atlas.ti [16] for further analysis. The qualitative descriptive analysis process was completed as per Miles et al [17], and inductive coding was allowed for codes and themes to emerge progressively.

After analyzing 25% of the content, a preliminary codebook [18] was developed as coding became redundant at that time. To ensure trustworthy findings and increase reliability [17], 2 investigators coded all of the data. First-cycle coding [17] was completed by 2 investigators (KP and EC or SK) to assign descriptive coding of the data using qualitative analysis software (Atlas.ti, version 8.4.2). The discrepancies in coding were discussed among the members and the codebook was adjusted as needed. Second-cycle coding was then completed to identify themes [17]. This study was submitted for review by the institutional review board at the University of Texas at Austin and deemed exempt given that this research project utilized information from publicly available datasets.

## Results

### General Description of the Data

A total of 526 individual mothers participated in the 20 posts and 912 related comments. The number of comments on the posts ranged from 4 to 152, with an average of 45.6 comments per post. Of the 20 posts, 6 included pictures. Table 1 includes a general description of each post and the total number of comments for each post.

The post that received the most likes (n=20) was a *Seattle Times* article about a doctor who linked hearing dysfunction to SIDS; if infants failed the screening, they would undergo a more thorough exam because they were more at risk than those who passed. The post that received the second highest number of likes (n=12) was a post from a mother who wanted to know who else “went against the recommendations” and let their babies sleep prone. Multiple posts received 0 likes and included the following topics: breathable bumpers, babies rolling prone, co-sleeping, and an infant who “hated*”* sleeping flat. Table 2 demonstrates the Facebook emotions and the number of comments that received such emotions. Of note, people who participated in *liking* a comment did not necessarily add a remark to the post. Therefore, the total number of participants in the analyzed conversations may have actually exceeded 526.

**Table 1:** General description of the Facebook posts and number of comments (in order, from most to least number of comments).

Comments, n	General content of original post (and ultimate conclusion, if known)
155	Mother attended a baby safety class and was told she should keep her baby in the same room for a year to prevent SIDS^a^. She was not planning on doing this and wanted to know what other moms’ opinions were on this topic
152	Mother is terrified of SIDS and is concerned because her husband would like to co-sleep. She would also like to co-sleep but is very anxious about it. She ultimately decided to co-sleep after receiving much input from other mothers
89	Mother has newborn who did not sleep well in bassinet and slept well in the Rock ‘n Play. She is not sure if the Rock ‘n Play is safe because of SIDS and asked if other mothers use it for sleep. After much input from other mothers, she chose not to use the Rock ‘n Play because her pediatrician advised against it
85	Mother cannot stop thinking about SIDS; she is terrified and asked other mothers how to cope with this stress. After much input from other mothers, she mentioned that she will likely proceed with purchasing a baby monitoring device
81	Mother wanted to know how many “went against the recommendations” and allowed their babies to sleep prone. Her mother and mother-in-law reassured her that prone sleeping is acceptable
42	Mother’s friend lost a baby to SIDS and she asked for suggestions on how to support her. After much input from other mothers, she thanked the group and shared her appreciation for the Facebook group
41	Mother is pregnant with her third baby and is scared of SIDS. She asked about other mothers’ experiences with the Owlet. After much input from other mothers, she mentioned that she will likely co-sleep but may consider placing the baby in a crib if she has reassurance from a monitor
36	Mother is terrified of SIDS and sleeps with baby in an in-bed bassinet and with a baby monitor. She asked if her feelings were normal
36	Mother shared link to an article discussing a study that found swaddling may cause SIDS. There was much input from mothers; some comments made light of the article and scrutinized scientific studies
31	Mother asked for input regarding co-sleeping. She fell asleep at the hospital with her baby and the hospital “freaked” her out because of SIDS, but she is considering co-sleeping because she breastfeeds
27	Mother asked other “tummy sleeper” mothers for reassurance. She quoted Dr Sears, who condones prone sleeping, and mentioned that other SIDS risks were low. After much input from other mothers, she mentioned that she found a swaddle on the web and that her baby was able to sleep supine
25	Mother asked for input about crib bumpers, mentioning that SIDS is no longer an issue because her baby is older than 6 months. After much input from other mothers, she mentioned that she will try bumpers and put stuffed animals in the crib corners (a suggestion from another mother)
24	Mother asked if other mothers let their babies sleep prone as her baby sleeps better this way; however, she is worried about SIDS
23	Mother asked if she can leave her baby prone once he or she rolls on his or her own; she is nervous because her baby is at the peak age for SIDS
20	Mother asked if she can leave her baby on stomach once he or she rolls on his or her own; she is following safe sleep guidelines and has a baby monitor but is still nervous about SIDS. After much input from other mothers (including recommendations for sleeping devices or monitors), she thanked the group and said that she felt better
18	Mother asked when she can stop worrying about SIDS because she would like to use crib bumpers and a blanket
11	Mother is concerned about SIDS after hearing “horror stories” and is considering the Owlet. She asked if anyone is selling one
6	Mother asked if other mothers’ babies will not sleep flat. Her baby is sleeping on a blanket on top of a nursing pillow, but she is scared of SIDS and knows flat is safer
6	Mother asked about breathable crib bumpers. She knows crib bumpers are controversial because of SIDS but wants to keep baby from hitting the crib and waking up. After much input, she thanked the group and says she will wait a bit longer but wanted to get input beforehand
4	Mother shared link to an article about a researcher who linked SIDS to children with audiologic problems

^a^SIDS: sudden infant death syndrome.

**Table 2:** Frequency of Facebook emotions per comments.

Facebook emotion	Comments receiving the emotion, n
1 like	241
2 likes	72
3 likes	36
4 likes	15
5 likes	12
1 love	45
2 loves	10
1 sad	10
2 sad	3

### Themes

In this analysis, the theme of social support emerged. Two types of social support were evident: informational and emotional support. The types of social support have been defined and modified by Cutrona and Suhr [19]. Informational support involves advice or suggestions, and emotional support involves sharing concern. Such categories of social support are applicable to web-based environments [20,21].

#### Informational Support

Informational support was evident in the following discussions among mothers: (1) asking questions about SIDS, infant sleep, or baby products; (2) sharing personal experiences of provider communication regarding SIDS or safe sleep; (3) sharing personal definitions or beliefs regarding SIDS or safe sleep (including discussions on vaccines and SIDS); and (4) sharing informational sources for SIDS or safe sleep. Overwhelmingly, the information shared demonstrated misinformation and inaccurate use of terminology.

##### Asking for Recommendations

Mothers openly asked for recommendations or feedback regarding infant sleep practices, use of baby products or monitoring devices, or other topics related to SIDS or safe sleep. Mothers asked for opinions about “what other moms’ opinions [were] on the subject” of prone sleeping, the use of the Rock ‘n Play, when to transition to a crib, crib bumpers, whether they should seek help for their anxiety about SIDS, or what monitor system to purchase (Owlet vs Snuza). Many of the responses involved information sharing of provider communication, personal definitions or beliefs, and information sources.

##### Provider Communication

Mothers shared information provided by their health care provider regarding SIDS, safe sleep, and the use of baby products. Some mothers shared their doctor’s knowledge of SIDS, such as the risk of SIDS being highest before 4 months of age or that the risk of SIDS from prone sleeping was “incredibly slim.” Another mother tried to distinguish co-sleeping from bed sharing; she mentioned that her doctor helped her understand that they were different. Some communication with providers endorsed safe sleep, whereas others condoned unsafe sleep. For instance, mothers mentioned that their pediatricians recommended supine sleeping and pacifier use and encouraged mothers not to use the Rock ‘n Play; however, other mothers mentioned that their pediatricians were “ok” with the Rock ‘n Play. One mother said that her pediatrician was “ok” with her use of a breathable crib bumper, and another mother said that her pediatrician endorsed prone sleeping for naptime, just not bedtime.

##### Personal Definitions or Beliefs

Mothers also shared information by providing their own personal definitions or beliefs regarding SIDS or safe sleep. There were approximately 80 personal definitions or beliefs shared, some of which were accurate and some were not. Some mothers felt the risk of SIDS was exaggerated; one mother did her “own research” and said that SIDS is actually not common as only “2000” infants die yearly. Another mother mentioned that “they are overzealous about the SIDS thing,” whereas another mother agreed that it is very rare. Similarly, another mother, who self-identified as a pediatric provider, said that most babies were not at risk for SIDS if they did not have other risk factors such as smoking, neurological issues, or vaccines. One mother even expressed anger with the Back to Sleep campaign—she felt the campaign caused flat heads and developmental delays. There was also confusion about SIDS versus suffocation death and co-sleeping versus bed sharing. One mother said that co-sleeping was not a risk for suffocation, especially if one was following safe bed-sharing guidelines. Many mothers felt that that co-sleeping was “ok” if done safely and that SIDS had “nothing to do with bed sharing.” One mother mentioned that “statistically it is safe for breastfed babies to co-sleep.” Although many of the shared definitions and beliefs were inaccurate, including a long discussion of how vaccines cause SIDS, some mothers shared accurate information. For instance, some mothers commented that SIDS can occur anywhere and that co-sleeping is a risk for suffocation. Other mothers explained ways to prevent SIDS: keeping the house cool, use of a pacifier, no blankets, not smoking, and placing the baby supine. Finally, some mothers disagreed that vaccines cause SIDS.

##### Information Sources

Mothers shared information sources with other mothers, as it related to SIDS or safe sleep. The list of information sources is shown in Table 3. Mothers also shared links to baby products or monitoring products to facilitate purchases.

**Table 3:** List of information sources shared or discussed in the Facebook group (in alphabetical order).

Information sources	Direct link or sources	Descriptions
AAP^a^	Not provided	Not provided
AAP press release	Link: https://www.aap.org/en-us/about-the-aap/aap-press-room/Pages/Bed-Sharing-Remains-Greatest-Risk-Factor-for-Sleep-Related-Infant-Deaths.aspx	The press release was about a study published in *Pediatrics* titled *Bed-sharing remains greatest risk factor for sleep-related infant deaths*
Babybargains.com	Link: www.Babybargains.com	From the website: “We obsess over baby gear . . . so you don’t have to. Baby Bargains has one mission: help you find the best gear for your baby with unbiased reviews by experts with 20 years of experience”
Baby Safety Academy	Not provided	Mom shared information from attending this class: “Infants should sleep in the same room as the parents for the first year of life”
Book: *Babywise*	Not provided	Not provided
Book: *Bearing the Unbearable*	Not provided	Not provided
Biologically Normal Infant Sleep	Facebook group	Per the mother who shared this source, this group had information on “safe co-sleeping” based on Dr James McKenna
Compassionate Friends	Online support group for parents who have lost a child	Not provided
Dr Sears	Not provided	Per the mother who shared this source, Dr Sears says the following: “The front-sleeping risk factor for SIDS^b^ doesn’t mean that you should worry every time you place your baby down to sleep. Just be sure to place your baby to sleep on a safe bedding surface. After all, over 99.9 percent of tummy-sleeping infants wake up every morning”
Ezinearticles.com	Link: https://ezinearticles.com/?Preventing-Tragedies---Moving-Air-From-a-Fan-is-All-You-Need-to-Prevent-SIDS%3F&id=1650718	The article was titled *Preventing tragedies - Moving air from a fan is all you need to prevent SIDS*. The article cited a study by Kaiser Permanente, published in *Archives of Pediatrics and Adolescent Medicine*, finding that fan use can prevent SIDS
Family Sleep Institute	Link: https://familysleepinstitute.com/position-statements-2/	The position statement discussed the update in safe sleep guidelines and provided a link to Charlie’s Kids (www.charlieskids.org) for further guidance on safe sleep
Gavin’s Gift of Grace	Facebook group	Not provided
Google	Not provided	Not provided
Gracie Faith SIDS Prevention Program Inc	Not provided	Not provided
Hand to Hold	Support group for parents who have lost a child	Not provided
*New York Times*	Link: https://well.blogs.nytimes.com/2016/05/09/swaddling-may-increase-the-risk-of-sids/	The article was about a study published in *Pediatrics*, finding that swaddling may contribute to SIDS
New Zealand study	Not provided	Per the mother who shared this source, a study in New Zealand found that toxins in mattresses contribute to SIDS
*NPR^c^*	Link: https://www.npr.org/sections/health-shots/2017/06/05/531582634/babies-sleep-better-in-their-own-rooms-after-4-months-study-finds	The article was about a study published in *Pediatrics*, finding that babies sleep better in their own rooms after 4 months
Rhett Sullivan Foundation	Link: https://rhettsullivan.org	From the website: the mission is to help “families who experience unexpected early child loss”
Safe Infant Sleep - Evidence-Based Support Group	Facebook group	Not provided
Safe Sleep and Baby Care - Evidence Based Support	Facebook group	Not provided
*Seattle Times*	Link: https://www.seattletimes.com/seattle-news/health/one-seattle-childrens-doctor-thinks-he-close-to-stopping-sids/	The article is about a doctor linking audiology problems to SIDS
Book: *Sweet Sleep: Nighttime and Naptime Strategies for the Breastfeeding Family*, written by La Leche League International and others	Link: https://books.apple.com/us/book/sweet-sleep/id813166216	From the website: “Research finds that most breastfeeding mothers do sleep with their babies at some point and preparing for bedsharing is safer than accidentally falling asleep together”
The Pediatric Insider	Link: https://pediatricinsider.wordpress.com/2015/05/28/swings-slings-and-car-seats-are-not-for-sleeping/	The article, written by a pediatrician, advises against using swings, slings, and car seats for sleeping. The blog referenced an article published in *Pediatrics* titled *Hazards associated with sitting and carrying devices for children two years and younger*

^a^AAP: American Academy of Pediatrics.

^b^SIDS: sudden infant death syndrome.

^c^NPR: National Public Radio.

#### Emotional Support

Emotional support was evident in the following discussions among mothers: (1) encouraging each other to “do what’s best for you or your family,” (2) telling each other that “it will get better with time,” (3) stories of infant or child death, (4) expressions of maternal anxiety about SIDS, and (5) general comments that relayed support.

##### Do What’s Best

Mothers offered emotional support by encouraging each other to do what was best for their family when talking about SIDS or safe sleep, regardless of the infant sleep environment or use of baby products. For example, it was common for a mother to comment “I think you need to do whatever you are comfortable with” or “you have to do whatever you feel comfortable with because if not, you’ll never sleep” regarding infant sleep environments. Many mothers supported following a “mother’s instinct” or “mother’s gut.” For example, one mother said “research, educate yourself, and trust your mama gut” when discussing how to help a new mother ease her worries about SIDS. Another mother commented: “whatever you choose to do will be right, because you are the mom. Your instincts will most always be correct.” There was also a focus on doing what was best for each family, despite what the safe sleep recommendations might be. For instance, it was common to see mothers comment “I think every family is different and not everything works for everyone” or “I think whatever you feel is right for you and your baby, just because it’s recommended doesn’t mean you have to follow.” One mother addressed the changing infant sleep recommendations, saying “…info changes all the time and we should all do what works for us and our baby.” However, some mothers did caution about the support and advice being given. For example, one mother said, “do what works best for you but keep in mind you’re asking for opinions and opinions do not change the research or statistics.”

##### It Gets Better With Time

Similar to the emotional support for mothers to do what was best for their family, emotional support was also prevalent in discussions of how worrying about SIDS gets better with time. Mothers reassured each other that the “worry of SIDS” was normal and that it would get better over time. One mother celebrated when her infant turned 4 months old and encouraged another mother that with time, she will realize “wow. I haven’t thought about SIDS in weeks.” Another mother said that her concerns subsided eventually, even though she still found herself standing over the crib at times.

##### Stories of Death

Emotional support was also prevalent in conversations surrounding stories of infant or child death. These conversations included mothers asking how to support mothers who lost an infant or child. For example, when discussing how to support a mother who had recently lost an infant to SIDS, one mother said, “my friend lost her daughter to SUIDS and talking about her daughter makes her happy.” Other mothers shared stories of infant or child death, including infants who died from co-sleeping or from “sleeping in rock and plays.”

##### Maternal Anxiety

Emotional support was evident in conversations surrounding the concept of maternal anxiety related to SIDS. Mothers openly asked if their anxiety about SIDS was normal and how to cope with such feelings. One mother said that postpartum stress is “absolutely awful;” she explained that she cried daily for weeks and felt that this was normal. Many mothers shared stories of watching their baby sleep throughout the night, mentioning they were “terrified of SIDS”. Mothers replied by offering support, by encouraging the use of baby products (Owlet or Snuza), recommending that the mother speak with a health care provider, or normalizing such feelings. For instance, it was common for a mother to say the Owlet was the “only way I [could] sleep…and helped my anxiety.”

##### Supportive Comments

Finally, emotional support was embedded within conversations around SIDS or safe sleep when mothers frequently shared supportive or encouraging comments. There were approximately 100 comments demonstrating support, evident in conversations of various topics. It was common to read “Good luck, mama,” “I was right there with you,” or “Hang in there.” Mothers demonstrated unity in feeling anxiety about SIDS, encouraged each other to not worry so much, and to get some sleep. Often, these words of encouragement were accompanied by emojis, such as hearts or kisses.

### Information Sources

Of the 20 main posts, 4 included references to an information source. Of those, the following were mentioned: *Seattle Times*, *Time Magazine*, Dr Sears, and Baby Safety Academy. It is worth noting that the American Academy of Pediatrics (AAP) was mentioned at least nine times throughout the posts and related comments. Table 3 lists the information sources that were shared in the posts and related conversations, with a brief description or direct link, if provided by the participant.

### Parental Practices of Infant Sleep

Of the 20 main posts, 12 involved infant sleeping practices. Of these, 2 were considered safe sleep environments; both of these posts were from mothers inquiring about what to do once their infant began rolling onto their stomach. Of the 12 posts that involved infant sleeping practices, 10 were considered unsafe practices. These included discussions about the following infant sleep practices: co-sleeping (2), sleeping on a nursing pillow (1), prone sleeping (3), crib bumpers (3), and the Rock ‘n Play (1). In analyzing the data, it became apparent that parental practices of infant sleep are best understood on a continuum rather than as a black and white matter. Infant sleep practices are surprisingly complex, and mothers adjust to the baby’s needs as well as their own family’s needs.

#### Unsafe Sleep

Many mothers changed sleep practices based on the infant and the needs of the family. For instance, many mothers did not bed-share with their first child, but chose to bed-share with their second. One mother encouraged other mothers to “listen to [their] instincts.” Another mother said that it just depends on the “family dynamic.”

Many mothers were aware of safe sleep recommendations but chose to practice unsafe sleep. For example, one mother encouraged others to join an evidenced-based group on safe sleep but mentioned that she bed shared with her first child and might with her second. There was also confusion around safe sleep recommendations and infant sleep practices. For example, one mother bed shared with her children, but only for the first few months before transitioning them to a crib, because “SIDS is less likely if they’re in bed.” Many mothers also allowed infants to sleep prone for nap time, but not at nighttime. One mother had 2 of her children sleep in the Rock ‘n Play and her third in a DockATot; she wanted them next to her, but in their own safe space, because she was “SIDS traumatized.”

Within conversations surrounding such practices, there was also evidence of motivation for unsafe sleep practices. One mother chose to bed-share because she had a cesarean section and it was a “chore” to walk to a crib. Many mothers chose to bed-share because their babies had reflux and they wanted to “be right next to [the baby] if anything happened.” Some mothers simply believed that their baby was safer next to them in bed. Many mothers “didn’t plan to co-sleep” but had a baby that would only sleep in bed with the mother or had choking because of reflux. Another mother allowed her infant to sleep in bed on a pillow because the mother could not rest “without [the baby] being near,” despite knowledge that such practices were not recommended. Mothers also explained that it was not feasible for infants to sleep in their bedrooms for the recommended 6 to 12 months because of personal difficulty sleeping and returning to work.

Among discussions of unsafe sleep practices were elements of confidence in such practices. Mothers felt confident they would “wake up the moment anything [happened]” or mentioned that they would never roll onto their infant when bed sharing. Another mother who was bed sharing explained that she was not worried about suffocation as the blanket did not reach the infant’s head and that she and her husband do not move throughout the night.

#### Safe Sleep

Although many comments described unsafe sleep environments, some mothers encouraged safe infant sleep environments. For instance, in responding to a mother who asked for advice about her infant who preferred to sleep prone, whereas many mothers endorsed this practice and recommended the Rock ‘n Play, one mother commented “SIDS is most common at 3 months old. Back is best.” Other mothers commented that the mother was taking a significant risk, and some explained why the mother should follow the “AAP safe sleep guidelines.”

### Sleeping Devices or Sleep Monitoring Devices

Discussions surrounding infant sleeping devices or monitoring devices permeated many conversations regarding SIDS or safe sleep. Mothers asked specific questions about the use of infant monitoring devices or sleep devices and encouraged other mothers to use devices to cope with their fear of SIDS. Of the 20 main posts, 10 involved the topic of sleeping devices or monitors. The breakdown of the sleeping device or sleep monitoring devices discussed in the original 20 posts are displayed in Table 4. Throughout all of the data analyzed, the device most commonly mentioned was the Owlet monitor, which was mentioned 112 times. The Snuza monitor was mentioned 53 times and the Angelcare monitor, 5 times. The Rock ‘n Play was mentioned 26 times and the DockATot, 4 times.

**Table 4:** Sleeping device or sleep monitoring device mentioned in main posts.

Sleeping devices or sleep monitoring devices	Posts, n
Crib bumpers	4
Owlet monitor	2
In-bed bassinet	1
Nursing pillow	1
Rock ‘n Play	1
Mimo Monitors	1

Mothers frequently mentioned that sleep monitoring devices provided “peace of mind” and used Facebook as a marketplace to buy and sell such devices. It was common for mothers to ask other mothers about their experience with the Owlet; one mother was considering purchasing one to “ease my mind… [and] help me to sleep better at night instead of always getting up to check that they’re still breathing.” Many responses encouraged the use of sleep monitoring devices. For example, many mothers said, “Owlet is my life” or “Owlet helped my SIDS anxiety” and expressed that the Owlet was one of the necessities of being a mom. For instance, one mother said that she could not live without the Owlet, diapers, and clothing. In comparison to many mothers recommending sleep monitoring devices, there were few mothers who advised against the use of sleep monitoring devices. One mother explained that the false alarms caused her more distress and advised against using the Owlet. Another mother said that it would be “one more thing to obsess over.”

There were also frequent conversations about other baby products. The Rock ‘n Play was by far the most frequently discussed device; however, mothers also discussed the HALO SleepSack, Merlin’s Magic Sleepsuit, DockATot, Love To Dream SWADDLE UP, Woombie, Snuggle Nest, etc. These discussions suggested strategies for improving infant sleep and either encouragement for use for a safer option (vs the Rock ‘n Play) or sometimes encouragement of use despite the known risk of SIDS.

### Provider Communication

In the dataset, there were approximately 30 mentions of health care provider communication regarding SIDS. Of the 20 original posts, 2 included health care providers and involved provider communication. These 2 examples include a mother who described how the hospital “freaked” her out when she fell asleep with the baby in the hospital bed and another mother who asked about prone sleeping—as doctors used to recommend this practice. Other conversations included pediatricians recommending safe sleep practices and pediatricians condoning unsafe sleep practices. For example, one pediatrician “had no problem” with a baby sleeping in the Rock ‘n Play, and a self-identified pediatric nurse claimed that she had never heard of some of the safe sleep recommendations. However, when one mother told her pediatrician about her use of the Rock ‘n Play, she was informed of the risks of suffocation.

## Discussion

### Principal Findings

As suggested by Huo and Turner [12], studies on social media user perspectives are needed to guide the development of social media interventions. However, we believe this analysis guides future SUID prevention interventions beyond social media, providing guidance for health care providers, public health agencies, and health campaigns. Similar to prior social media analyses [22,23], this analysis has revealed new findings regarding maternal perspectives of SIDS and safe sleep that are otherwise not discussed in the literature. This analysis also supports prior findings regarding parental practices of infant sleep and provider communication of SUID prevention.

#### Themes

Social media has been identified as a supportive environment in prior analyses and studies [15,24-26], which was consistent with the identified theme of support. Understanding the types of support, informational and emotional, provided among mothers and in this format is a new finding. This Facebook mother’s group promoted participant engagement and honesty about very personal health-related concerns, resulting in discussions about maternal anxiety, personal practices of infant sleep, and discussions about infant sleeping devices and sleep monitoring devices. This frankness among social media users has previously been discovered in discussions about health-related topics [23]. One implication of this finding is the potential of emulating this type of supportive environment in other settings. For instance, health care organizations could consider creating more supportive environments in our current health care system to promote open discussions with parents. Additionally, many of the comments and conversations in this analysis provided opportunities for correction of misinformation or discussions regarding options for safer infant sleep. Although mothers receive information via many different routes [13], social media remains incredibly popular [27] and is a platform where patients can receive and communicate health information. Since Facebook mother groups are available 24/7, and most health care providers are not, health care organizations should also rethink how services are structured. For instance, if a nurse or provider were truly accessible 24/7 for advice via a social media format, this could potentially influence and impact parental decisions and decrease the spread of misinformation that is otherwise widespread among social media sites [28].

#### Information Sources

The information sources shared among the mothers regarding SIDS or safe sleep demonstrated a variety of sources. We know that mothers obtain information from many different sources [13], and this study supports this. However, this study identified sources specific to SIDS and safe sleep. Consistent again with a Twitter analysis on SIDS and safe sleep [24], there was evidence of news media organizations, such as *National Public Radio*, being shared. However, a concerning finding was the sharing of informal and potentially inaccurate information sources, such as Dr Sears, other Facebook groups, and controversial books such as *Babywise*. Thus, shared sources were often inconsistent with the AAP recommendations. This is similar to the findings from a prior study of Google searches that identified information largely contradicting AAP recommendations of infant safe sleep [29]. The commonality of shared links for purchase of baby products was also worrisome given that many of the shared products are not considered safe for infant sleep nor recommended by the AAP [3]. Thus, health care providers may consider asking parents where they obtain health information to openly discuss and potentially correct any misinformation that is shared in that format.

#### Parental Practices of Infant Sleep

Another finding is that infant sleep practices are not straightforward; safe sleep and unsafe sleep are best described on a continuum. Mothers alter infant sleep practices based on the infant and family’s needs. Many mothers in the group were aware of safe sleep recommendations but chose not to follow them because they were not feasible. This finding supports a prior study’s conclusion that parental motivation to bed-share trumped known risks of unsafe sleep [30]. The discovery of mothers who are practicing unsafe and ever-changing infant sleep practices is also consistent with studies demonstrating parental practice of unsafe sleep [5,6,31,32] and the changing of infant sleep environments throughout the night [33]. In this Facebook group, some mothers endorsed safe sleep and recommended following the AAP guidelines; however, other mothers believed they were following the recommendations and were actually creating dangerous infant sleep environments. Safe sleep is seemingly complex and further complicated by the confusion surrounding definitions of SIDS and safe sleep. For instance, this analysis demonstrated wide confusion and inaccurate use of the terms *co-sleeping* and *bed sharing*. These findings imply that simpler terms should be used in public health campaigns and when educating families about SUID prevention. This finding also informs health communication; health communication about safe sleep and SUID prevention should use simple language, be direct, and be clear.

#### Sleeping Devices or Sleep Monitoring Devices

This study demonstrates the popularity and commonality of infant sleep monitoring devices and baby products; thus, this topic can no longer be ignored. This is similar to the findings from a Twitter analysis on SIDS and safe sleep, where conversations and advertising about such products were widespread [24]. It is worth noting that the literature is sparse regarding infant sleep monitoring products and baby products. Health care providers need to educate themselves on popular products, so they can effectively discuss this topic with parents. For example, it may be helpful for health care providers to directly ask parents “What devices are you using to help your baby sleep?” Public health agencies should also consider addressing such devices in campaigns and in health messaging.

#### Provider Communication

Furthermore, when discussing the topic of SUID and safe sleep, health care providers and public health agencies need to continue to provide accurate information on a consistent basis. Health care provider advice on safe sleep impacts parental decisions [11], and inconsistency has been demonstrated among safe sleep messaging provided by health care professionals [34,35]. As this study also supports inconsistency in health care provider communication about safe sleep, there is room for improvement. Instead of assuming parental knowledge of safe sleep guidelines and that families are following such guidelines, health care providers should encourage open and honest conversations about infant sleep practices that occur throughout the night and at naptime. Health care providers should also recognize the need to consistently and correctly share safe sleep recommendations with families and caregivers.

#### Maternal Anxiety

Finally, the prevalence of discussions surrounding the topic of maternal anxiety related to SIDS and how mothers reassured each other that these feelings were normal is concerning. Many mothers coped with these feelings by using baby monitoring devices or baby sleeping devices rather than taking precautionary measures to prevent SUID and sleep-related infant death. Persons with mental illness who openly share their stories and feelings offer insight into various illnesses and symptomology [36]; thus, this analysis may have actually identified mothers who have postpartum disorders. According to O’Hara and Wisner [37], the prevalence of major and minor depression in pregnancy and the postpartum period is 20%; early symptoms can be detected through screening, and early treatment is essential for the well-being of the mothers and children. Although some mothers shared that they had sought medical treatment for postpartum disorders, many did not. This topic raises the question of whether our health care system is successfully screening and identifying mothers who may need additional support and treatment for postpartum anxiety and depression.

### Limitations

Although this study provides new insights into maternal perspectives regarding SIDS and safe sleep, it is not without limitations. This particular Facebook mother’s group was selected because of the large number of participants; however, the views expressed may not represent potential opinions, concerns, and views of other Facebook mother group participants (or other mothers for that matter). Furthermore, demographic information of the participants was not obtained, limiting the generalizability of the findings. This analysis only included mothers; therefore, other perspectives (such as that of the father or other caregivers) were missed. Although many of the findings were consistent with a different social media analysis on SIDS and safe sleep [24], an analysis of another social media format may have resulted in contradictory findings. Additionally, social media language can be difficult to analyze; there are often typos, informal writing, or abbreviations that can make the analysis challenging [38]. Although measures were taken to prevent misinterpretation and the research team refrained from interpretation as much as possible, it is possible that some of the original content may have been misunderstood or misinterpreted. Finally, the participants often used safe sleep definitions inaccurately, potentially impacting the analysis. For instance, *co-sleeping*, *bed sharing*, and *room sharing* were often used interchangeably. The research team interpreted the meaning of the terms in the context used; however, it is possible that this was interpreted inaccurately.

### Conclusions

Despite such limitations, this analysis provides new information regarding maternal perspectives on SIDS and safe sleep: (1) a wide variety of information sources, (2) widespread utilization of infant sleep products and monitoring devices, and (3) maternal anxiety regarding SIDS. This study demonstrated confusion among the terminology commonly used in the medical community when speaking of SUID and safe sleep, implying that future communication should aim for simpler and clearer terms. Widespread practices of unsafe sleep and inconsistency in provider communication regarding SUID prevention are not new findings but emphasize the need for continued efforts in SUID education and prevention. Health care providers, health care organizations, and public health agencies should incorporate these findings in future research and health campaigns and when directly communicating with families about SIDS prevention and safe sleep. Furthermore, such organizations should consider using social media in their marketing efforts and actively engage in such formats to correct misinformation.

## Abbreviations

AAP: American Academy of Pediatrics
SIDS: sudden infant death syndrome
SUID: sudden unexpected infant death
